# Perception of the 2020 SARS-CoV-2 pandemic among medical professionals in Germany: results from a nationwide online survey

**DOI:** 10.1080/22221751.2020.1785951

**Published:** 2020-07-13

**Authors:** Pia Paffenholz, Arne Peine, Martin Hellmich, Stella V. Paffenholz, Lukas Martin, Mark Luedde, Miriam Haverkamp, Christoph Roderburg, Gernot Marx, Axel Heidenreich, Christian Trautwein, Tom Luedde, Sven H. Loosen

**Affiliations:** a Department of Urology, Uro-Oncology, Robot Assisted and Reconstructive Urologic Surgery, University Hospital Cologne, Cologne, Germany; b Department of Intensive Care Medicine and Intermediate Care, University Hospital RWTH Aachen, Aachen, Germany; c Faculty of Medicine, Institute for Medical Statistics and Computational Biology (IMSB), University Hospital Cologne, University of Cologne, Cologne, Germany; d Department of Cancer Biology and Genetics, Memorial Sloan Kettering Cancer Center, New York, NY, USA; e Louis V. Gerstner Jr. Graduate School of Biomedical Sciences, Memorial Sloan Kettering Cancer Center, New York, NY, USA; f KGP Bremerhaven, Bremerhaven, Germany; g Department of Infection Control and Infectious Diseases, University Hospital RWTH Aachen, Aachen, Germany; h Department of Gastroenterology/Hepatology, Charité University Medicine Berlin, Berlin, Germany; i Department of Medicine III, University Hospital RWTH Aachen, Aachen, Germany; j Division of Gastroenterology, Hepatology and Hepatobiliary Oncology, University Hospital RWTH Aachen, Aachen, Germany; k Clinic for Gastroenterology, Hepatology and Infectious Diseases, Medical Faculty, University Hospital Düsseldorf, Heinrich-Heine-University, Düsseldorf, Germany

**Keywords:** COVID-19, healthcare workers, personal protective equipment, PPE, nurses, burden

## Abstract

**Background:** The COVID-19 pandemic represents an unprecedented global challenge and implicates a wide range of burden on medical professionals. Here, we evaluated the perception of the COVID-19 pandemic among medical professionals in Germany.

**Methods:** A total of *n* = 2827 medical professionals participated in an online survey between 27 March and 11 April.

**Results:** While most participants stated that Germany was well prepared and rated the measures taken by their employer as positive, subgroup analyses revealed decisive differences. The preventive measures were rated significantly worse by nurses compared to doctors (*p* < 0.001) and by participants from ambulatory healthcare centres compared to participants from maximum-care hospitals (*p* < 0.001). Importantly, shortage of protective medical equipment was reported more commonly in the ambulatory sector (*p* < 0.001) and in East German federal states (*p* = 0.004). Moreover, the majority of health care professionals (72.4%) reported significant restrictions of daily work routine. Finally, over 60% of medical professionals had concerns regarding their own health, which were more pronounced among female participants (*p* = 0.024).

**Conclusion:** This survey may indicate starting points on how medical professionals could be supported in carrying out their important activities during the ongoing and future healthcare challenges.

## Introduction

On 12 March, the WHO declared the SARS-CoV2 outbreak originating from Wuhan, China a pandemic. As of 27 April, over 3 million cases have been confirmed globally and the estimated number of unknown cases is believed to exceed this number decisively [1]. The pandemic represents a global challenge for healthcare providers, patients and societies throughout the world. While the majority of COVID-19 patients presents with only mild symptoms such as fever, cough, myalgia or mild dyspnoea, up to 10% of patients, mostly elderly and patients with preexisting medical conditions, develop severe respiratory symptoms that require admission to the intensive care unit (ICU), mechanical ventilation or even extracorporeal membrane oxygenation (ECMO) therapy [2]. These patients are currently putting medical systems around the globe to the test and even medically highly developed countries struggle to provide sufficient medical care for all COVID-19 patients [3]. Based on reports from countries that were affected earlier by the COVID-19 outbreak, the German healthcare system was confronted with drastic measures, such as increasing intensive care capacities or postponing non-urgent clinic visits in order to prevent an overload of health care providers. These measures, together with extensive restrictions of public life, have shown a visible effect in terms of infection rates and COVID-19 mortality in Germany [1]. Nevertheless, reports about shortages of protective medical equipment (e.g. face masks) also arose in Germany. Moreover, data from Asia and other geographical regions report an enormous psychological burden especially on medical professionals [4–6]. With this nationwide online survey, we aimed at evaluating the perception of the SARS-CoV-2 pandemic among medical professionals in Germany in terms of general, work-related and personal aspects between 27 March and 11 April 2020.

## Materials and methods

### Data collection

Data collection took place between 27 March and 11 April 2020 on all days in the assessed timeframe. Participant acquisition was achieved through numerous communication channels, taking the heterogeneous access and technical capabilities of medical professionals into account: Survey access (internet link to the survey) was shared through the official communication channels (email distribution list for registered members) of various German medical societies (e.g. German respiratory Society), through email distribution list and the intranet of German hospitals as well as through distribution in social media. Due to this heterogeneous approach, a detailed evaluation of response rates was not feasible. The questions of the online survey were explicitly generated by the authors and were not based on existing standardized survey instruments (e.g. summarized in [5,6]). Survey data was acquired through a publicly accessible, web-based survey system (LimeSurvey, Version 3.22.10). The server infrastructure was hosted on an Apache web server (The Apache Software Foundation, USA) with location in Nuremberg, Germany and was reachable through the internet domain www.meinungsbild-corona.de. No downtime was observed during the acquisition period. Data storage was achieved using a MySQL database (My Structured Query Language, Oracle Corporation, Redwood City, USA). All participants agreed on the conditions of the survey and the publication of results before taking part.

### Statistical analysis

Data are given as percentage values of the respective group as well as in total numbers. Comparison of ordinal data was performed using Mann–Whitney-U-test for two groups and Kruskal–Wallis-test for more than two groups (both corrected for ties). Pairwise post-hoc analyses of significant results in the Kruskal–Wallis-test were performed by Mann–Whitney-*U*-test. In case of multiple pairwise comparisons, the level of significance was adjusted by Bonferroni correction. Thus, for comparisons between three groups a *p*-value of *p* < 0.017 and for comparisons between six groups a *p*-value of *p* < 0.003 was considered statistically significant. Otherwise, a *p*-value of *p* < 0.05 was used to determine statistical significance. Association of two categorical (nominal) variables was evaluated by Pearson’s chi-squared test. Correlation analyses were performed using point-biserial correlation (i.e. Pearson correlation coefficient) in case of a continuous and a binary variable and Spearman’s rank correlation in case of a continuous and an ordinal variable. Statistical analyses were performed with SPSS 23.0 (IBM Corp., Armonk, NY, USA). See supplementary material and method section for further details.

## Results

### Characteristics of the study population

A total of *n* = 2827 medical professionals participated in the online survey between 27 March and 11 April. In terms of the professional group, 65.6% were classified as doctors, 29.5% as nursing staff and 4.9% as others (e.g. psychotherapists, physiotherapists, occupational therapists). The median age of the study population was 42 years (range: 18–80 years). 51.1% reported to be female and 47.6% reported to be male. With respect to the work site, 43.8% of medical professionals were employees of a university hospital or maximum-care hospital, 26.5% worked at a regional hospital, 21.6% in an ambulatory healthcare centre or medical practice, 2.1% in a private clinic, 1.6% in rehabilitation clinic and 3.9% worked in other healthcare fields (e.g. ambulatory nursing service). Work environment was reported as follows: 10% outpatient clinic, 26.5% standard care ward, 17.2% intensive care unit (ICU), 14.2% operating room and 5.2% diagnostics. Table 1 provides a detailed overview of the study population.

**Table 1.** Characteristics of study population.

Characteristics	Study population
Number of participants	*n* = 2827
Age (years, median and range)	42 [18–80]
Gender (%) Female Male Diverse no answer	51.1 47.6 0 1.3
Professional group (%) Doctors Nursing staff Others (e.g. psychotherapists, physiotherapists, occupational therapists)	65.6 29.5 4.9
Work site (%) University Hospital/maximum-care hospital Regional hospital Ambulatory healthcare centre/medical practice Private clinic Rehabilitation clinic Others (e.g. ambulatory nursing service) No answer	43.8 26.5 21.6 2.1 1.6 3.9 0.5
Work environment (%) Outpatient clinic Standard care ward Intensive care unit Operating room Diagnostics Not applicable (e.g. ambulatory sector)	10.0 26.5 17.2 14.2 5.2 26.8
Federal state of the work site (%) East German federal states (inlc. Berlin) West German federal states	89.6 10.4
Timeframe of online survey	27.03.2020 to 11.04.2020

### General perception of the SARS-CoV-2 pandemic among medical professionals in Germany

Medical professionals were first asked about the degree of perceived threat due to the COVID-19 outbreak. Most participants reported a moderate level of threat (50.2%, Table 2). Interestingly, the level of threat significantly differed between participants’ characteristics. Female participants reported a higher level of threat compared to male participants (*p* < 0.001) and medical doctors reported a lower level of threat compared to the nursing staff (*p* < 0.001), while the level of threat did not significantly differ between work sites (*p* = 0.093). There was no correlation between the participants’ age and the perceived level of threat level (*r*_S_ = −0.030, *p* = 0.119).

**Table 2.** General perception of the SARS-CoV-2 pandemic among medical professionals in Germany.

	Not at all (1)	Hardly (2)	Moderately (3)	Strongly (4)	Very strongly (5)	*p*-value
**How threatened do you feel by the COVID-19 pandemic?**	2.9% (82)	18.2% (515)	50.2% (1419)	23.4% (661)	5.3% (150)	
Female Male	1.9% (28) 4.0% (54)	15.1% (218) 21.5% (289)	50.5% (730) 50.0% (673)	26.5% (383) 20.0% (269)	6.0% (87) 4.5% (60)	< 0.001
Medical doctors Nursing staff Others	3.1% (57) 2.6% (22) 2.2% (3)	19.5% (362) 14.8% (123) 21.6% (30)	51.5% (955) 47.5% (396) 48.9% (68)	21.5% (400) 27.5% (229) 23.0% (32)	4.4% (81) 7.6% (63) 4.3% (6)	< 0.001 0.931
**Do you fear that past health policy decisions will have a negative impact on the COVID-19 pandemic in Germany?**	1.1% (31)	7.4% (201)	20.6% (562)	39.7% (1083)	31.2% (850)	
Medical doctors Nursing staff Others	0.9% (16) 1.4% (11) 3.2% (4)	8.3% (150) 4.8% (38) 10.3% (13)	22.5% (407) 15.7% (125) 23.8% (30)	40.4% (729) 39.2% (312) 33.3% (42)	27.9% (503) 38.9% (310) 29.4% (37)	< 0.001 0.428
Intensive care Unit (ICU) Other work environments (outpatient clinic, standard care ward, operating room, diagnostics)	1.3% (6) 1.2% (18)	5.8% (27) 7.5% (115)	19.3% (90) 20.1% (308)	36.4% (170) 40.8% (627)	37.3% (174) 30.4% (467)	0.019
	Very poor (1)	Poor (2)	Neutral (3)	Good (4)	Very Good (5)	
**How well prepared do you think Germany is for the COVID-19 pandemic?**	6.5% (183)	31.2% (883)	24.7% (698)	34.1% (963)	3.5% (100)	
Medical doctors Nursing staff Others	5.0% (93) 10.3% (86) 2.9% (4)	29.8% (552) 35.3% (294) 26.6% (37)	24.0% (446) 25.5% (212) 28.8% (40)	37.1% (688) 26.4% (220) 39.5% (55)	4.1% (76) 2.5% (21) 2.2% (3)	< 0.001 0.520
University hospital/maximum-care hospital Regional hospital Ambulatory healthcare centre/medical practice Private clinic Rehabilitation clinic Others (e.g. ambulatory nursing service)	5.1% (63) 6.1% (46) 8.0% (49) 6.7% (4) 6.7% (3) 14.4% (16)	27.5% (340) 30.8% (231) 37.8% (231) 30.0% (18) 28.9% (13) 39.6% (44)	24.6 (304) 25.5% (191) 24.1% (147) 30.0% (18) 15.6% (7) 24.3% (27)	38.7% (479) 34.7% (260) 27.2 (166) 26.7% (16) 40% (18) 20.7% (23)	4.2% (52) 2.8% (21) 2.9% (18) 6.7% (4) 8.9% (4) 0.9% (1)	0.009 < 0.001 0.294 0.655 < 0.001
**How do you rate the increasing restrictions in public life?**	1.1% (29)	3.8% (102)	5.1% (138)	31.9% (856)	58.1% (1562)	
	1 Month (1)	3 Month (2)	6 Month (3)	12 Month (4)	>1 year (5)	
**How long will it take until public life mainly normalizes in Germany?**	1.3% (36)	20.6% (583)	40.9% (1155)	17.2% (486)	20.1% (567)	
	<1% (1)	1–3% (2)	3–5% (3)	5–10% (4)	>10% (5)	
**What percentage of infected people will die from COVID-19 infection in Germany?**	21.4% (606)	51.9% (1467)	19.4% (549)	6.4% (180)	0.9% (25)	
Medical doctors Nursing staff Others	27.1% (505) 10.0% (83) 12.9% (18)	55.1% (1023) 44.1% (367) 55.4% (77)	14.3% (266) 30.3% (252) 22.3% (31)	3.1% (58) 13.3% (111) 7.9% (11)	0.2% (3) 2.4% (20) 1.4% (2)	<0.001 <0.001

Notes: In case of multiple pairwise comparisons, the level of significance was adjusted by Bonferroni correction. A *p*-value of 0.017 for three groups and 0.003 for six groups was considered statistically significant.

**Figure 1.**
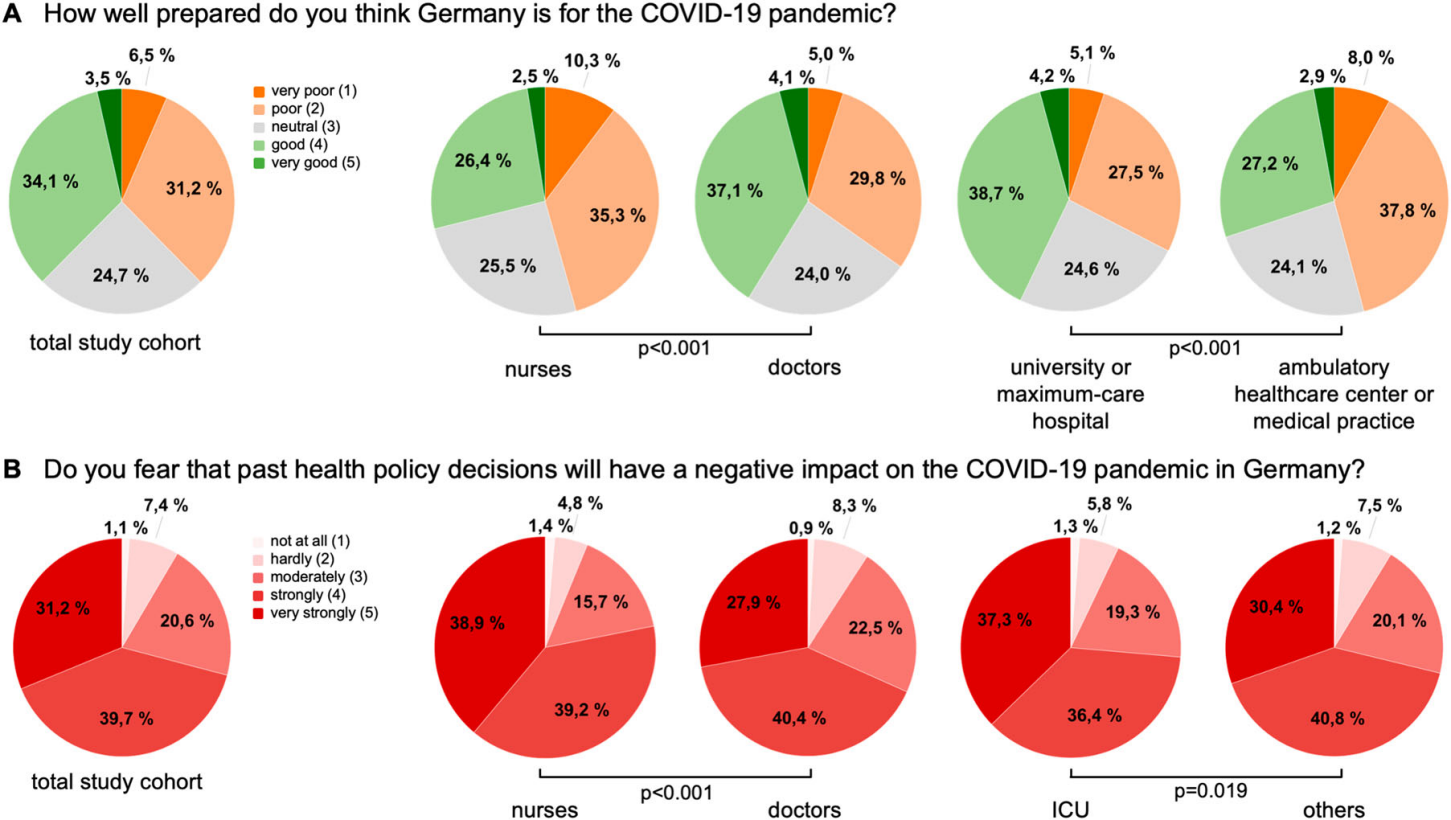
Preparations of Germany for the COVID-19 pandemic. (A) The nursing staff state that Germany is significantly worse prepared compared to doctors (*p* < 0.001). Germany’s preventive measures are rated significantly better at university and maximum-care hospitals compared to ambulatory healthcare centers and medical practices (*p* < 0.001). (B) Compared to doctors, nurses fear a more negative impact of past healthcare policy decisions on the COVID-19 pandemic in Germany (*p* < 0.001). Participants working on an ICU assume a more negative impact compared to participants from other working environments (*p* = 0.019).

Next, we asked medical professionals about their assessment of how well-prepared Germany was for this pandemic as well as their assessment of the current measures of public restriction imposed by the federal government. Most participants stated that Germany was well prepared (37.1%) and rated the public restrictions as “very good” (58.1%, Table 2). However, we observed decisive differences particularly regarding the professional group and work site. As such, the nursing staff felt that Germany was significantly worse prepared compared to doctors (*p* < 0.001, Table 2). Germany’s preventive measures were rated significantly better at university and maximum-care hospitals compared to ambulatory healthcare centres and medical practices (*p* < 0.001, Figure 1(A), Table 2). When asked whether or not past healthcare policy decisions (e.g. number of nurses on the ICU) might have a negative impact on the COVID-19 pandemic in Germany, most healthcare professionals (39.7%) feared a “strong” negative impact of former political decisions on the course of COVID-19 in Germany (Table 2). Again, this impression was more prominent in the nursing sector (*p* < 0.001, Figure 1(B), Table 2) and was consistent among the different work sites (*p* = 0.157). However, participants working on an ICU feared a significantly higher negative impact compared to participants from other working environments (*p* = 0.019, Figure 1(B), Table 2).

Subsequently, we asked medical professionals about their general future outlook with respect to the COVID-19 outbreak in Germany. When asked how long it will take until daily life in Germany will mostly be normalized, most participants (40.9%) estimated “6 months” (Table 2). Regarding the estimated maximum cumulative number of SARS-CoV-2 infections in Germany, the most prominent answer was “1–10 million” (23.1%). Finally, the majority of participants (51.9%) assumed an overall mortality of COVID-19 in Germany between 1 and 3% (Table 2). This estimation was significantly higher among participants from the nursing sector compared to doctors (*p* < 0.001, Table 2).

### Evaluation of work-related aspects within the German health care system during the COVID-19 pandemic

We subsequently concentrated on work-related aspects within the German health care system. Participants were first asked whether or not their daily work routine changed due to the COVID-19 outbreak. Here, more than 80% of participants stated that their daily work routine has “strongly” (41.9%) or even “very strongly” (40.0%) changed (Table 3). Doctors reported a higher level of change compared to the nursing staff (*p* = 0.008). Next, we surveyed changes in the health system’s operating procedures in terms of e.g. the delay of non-critical medical procedures to save medical resources for COVID-19 patients. The majority of healthcare professionals (72.4%) reported significant changes at their institution with “almost no elective procedures except for urgent ones such as tumor surgery” (Table 3). When comparing different work sites, ambulatory healthcare centres/medical practices and “other work sites” (e.g. ambulatory nursing services) reported less severe cuts regarding operating procedures (both *p* < 0.001, Table 3).

**Table 3.** Evaluation of work-related aspects within the German health care system during the COVID-19 pandemic.

	Not at all (1)	Hardly (2)	Moderately (3)	Strongly (4)	Very strongly (5)	*p* -value
**Has your daily work routine changed because of the COVID-19 outbreak?**	0.4% (11)	3.1% (86)	14.6% (400)	41.9% (1145)	40.0% (1093)	
Medical doctors Nursing staff Others	0.4% (8) 0.3% (2) 0.8% (1)	2.3% (42) 4.8% (38) 5.5% (6)	14.5% (262) 14.4% (115) 18.0% (23)	41.1% (744) 44.4% (354) 36.7% (47)	41.6% (753) 36.2% (289) 39.8% (51)	0.008 0.271
**Do you expect the COVID-19 pandemic to increase the financial value of your professional group?**	30.3% (827)	38.1% (1039)	15.6% (427)	9.2% (251)	6.8% (185)	
Medical doctors Nursing staff Others	35.0% (633) 21.0% (167) 21.4% (27)	40.8% (736) 32.4% (258) 35.7% (45)	14.7% (266) 16.3% (130) 24.6% (31)	6.4% (115) 15.2% (121) 11.9% (15)	3.1% (56) 15.2% (121) 6.3% (8)	< 0.001 < 0.001
	Not at all (1)	Hardly (2)	Significantly (3)	Completely (4)		*p* -value
**To what extent is the routine operation of your institution restricted due to the COVID-19 pandemic?**	2.7% (73)	13.0% (355)	72.4% (1974)	11.9% (323)		
University hospital/maximum-care hospital Regional hospital Ambulatory healthcare centre/medical practice Private clinic Rehabilitation clinic Others (e.g. ambulatory nursing service)	2.3% (28) 1.4% (10) 2.4% (14) 1.7% (1) 4.5% (2) 16.5% (16)	9.2% (110) 8.7% (63) 20.5% (122) 25.4% (15) 29.5% (13) 28.9% (28)	76.8% (916) 78.2% (568) 66.6% (396) 59.3% (35) 47.7% (21) 33.0% (32)	11.6% (138) 11.7% (85) 10.6% (63) 13.6% (8) 18.2% (8) 21.6% (21)		0.439 < 0.001 0.045 0.038 < 0.001
	Never (1)	Only once (2)	Only for a short time (3)	Regularly (4)	Continuously (5)	*p* -value
**Was there a shortage of consumables (e.g. face masks or protective gowns) in your institution at one time to protect against SARS-CoV-2?**	21.9% (596)	7.0% (191)	27.5% (749)	20.9% (569)	22.7% (618)	
University hospital/maximum-care hospital Regional hospital Ambulatory healthcare centre/medical practice Private clinic Rehabilitation clinic Others (e.g. ambulatory nursing service)	26.5% (316) 25.7% (186) 9.2% (55) 25.4% (15) 18.2% (8) 14.4% (14)	8.0% (95) 7.0% (51) 5.0% (30) 10.2% (6) 9.1% (4) 4.1% (4)	30.9% (368) 31.2% (226) 16.1% (96) 28.8% (17) 36.4% (16) 24.7% (24)	18.1% (215) 21.4% (155) 26.5% (158) 16.9% (10) 13.6% (6) 22.7% (22)	16.5% (197) 14.8% (107) 43.1% (257) 18.6% (11) 22.7% (10) 34% (33)	0.663 < 0.001 0.875 0.312 < 0.001
East German federal states West German federal states	22.0% (537) 20.6% (59)	7.2% (176) 5.2 (15)	28.0% (683) 23.1% (66)	21.1% (515) 18.9% (54)	21.6% (526) 32.2% (92)	0.004
	Very negative (1)	Negative (2)	Neutral (3)	Positive (4)	Very positive (5)	*p* -value
**How do you rate the measures taken by your employer against COVID-19?**	3.7% (101)	15.7% (429)	25.0% (680)	40.3% (1097)	15.3% (418)	
University hospital/ maximum-care hospital Regional hospital Ambulatory healthcare centre/medical practice Private clinic Rehabilitation clinic Others (e.g. ambulatory nursing service)	3.3% (39) 4.3% (31) 3.0% (18) 3.4% (2) 6.8% (3) 8.1% (8)	15.7% (187) 17.5% (127) 10.5% (62) 25.4% (15) 20.5% (9) 25.3% (25)	22.7% (271) 26.6% (193) 27.9% (165) 18.6% (11) 27.3% (12) 22.2% (22)	40.6% (485) 39.3% (285) 44.1% (261) 35.6% (21) 27.3% (12) 32.3% (32)	17.8% (212) 12.4% (90) 14.5% (86) 16.9% (10) 18.2% (8) 12.1% (12)	0.001 0.889 0.278 0.164 0.001

Notes: In case of multiple pairwise comparisons, the level of significance was adjusted by Bonferroni correction. A *p*-value of *p* = 0.017 for three groups and *p* = 0.003 for six groups was considered statistically significant.

**Figure 2.**
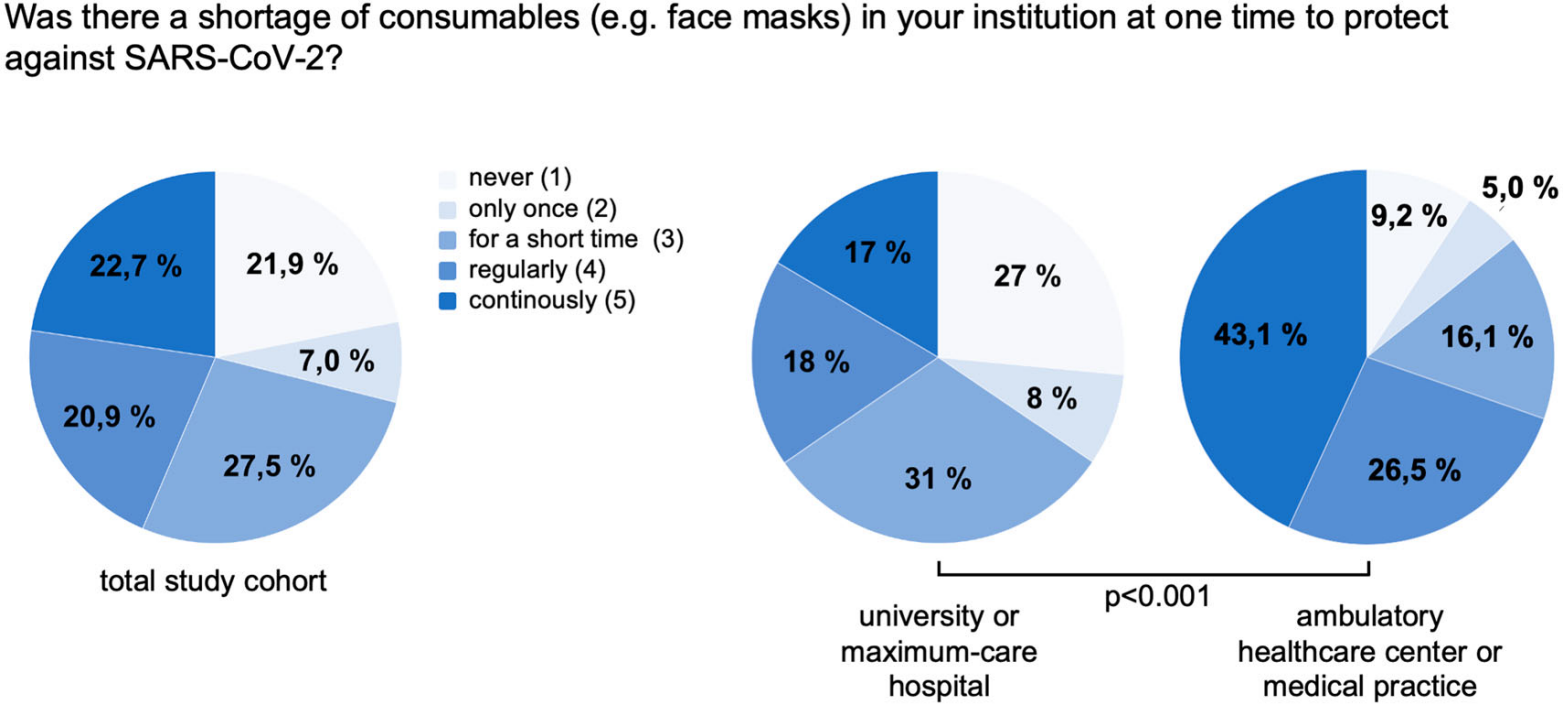
Shortage of medial protective equipment. According to the statement of medical professionals in Germany, shortage of medical protective equipment is significantly more common in ambulatory healthcare centres/medical practices compared to university hospitals (*p* < 0.001).

We next evaluated if shortage of consumables such as medical protective equipment is also of relevance within the German healthcare system. Interestingly, while most participants (27.5%) reported only short-term shortages, over 40% of medical professionals in Germany stated that there was a regular (18.1%) or even permanent (16.5%) shortage of consumables at their institution (Table 3). Most importantly, the outpatient healthcare sector was particularly affected because shortage of medical protective equipment was significantly more common in ambulatory healthcare centres/medical practices compared to e.g. university hospitals (*p* < 0.001, Figure 2, Table 3) or regional hospitals (*p* < 0.001). Moreover, a shortage of consumable materials was more frequently observed in the geographical region of the East German federal states (incl. Berlin) compared to the West German federal states (*p* = 0.004, Table 3).

When asked to evaluate the measures taken by their employer with respect to the COVID-19 outbreak, most medical professionals (40.3%) rated the measures as “positive” (Table 3). However, employees of regional hospitals as well as “other work sites” (e.g. ambulatory nursing service) rated their employers’ measures significantly worse compared to e.g. university and maximum-care hospitals (both *p* = 0.001, Table 3). Furthermore, 47.2% of all participants reported that their employer had provided a specific COVID-19 training to be better prepared for the pandemic. Importantly, COVID-19 training was more frequently offered at university/maximum-care (51.1%) or regional hospitals (54.0%) compared to ambulatory healthcare centres/medical practices (35.8%, both *p* < 0.001, not shown in Table 3). In addition, COVID-19 training was significantly more frequently offered to doctors (50.9%) compared to the nursing staff (39.3%, *p* < 0.001, not shown in Table 3). Finally, we evaluated whether or not medical professionals expected the COVID-19 pandemic to increase the financial value of their professional group e.g. through future health policy decisions. The most frequent answer (38.1%) was “hardly” and over 30% of participants did not expect any financial benefit at all (Table 3). Of note, doctors significantly less frequently expected an increase in their income compared to both the nursing staff (*p* < 0.001) and “others” (*p* < 0.001, Table 3).

### Impact of the COVID-19 pandemic on personal aspects of medical professionals in Germany

**Figure 3.**
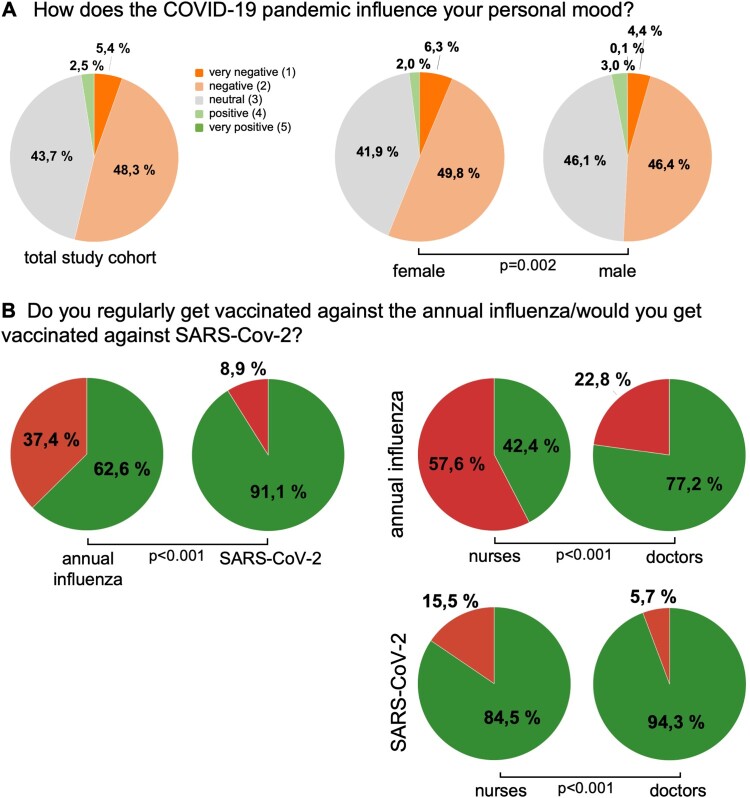
Personal impact of the COVID-19 pandemic. (A) The personal mood of female participants is more negatively influenced by the COVID-19 pandemic compared to males. (B) The willingness to be vaccinated against SARS-CoV-2 – once a clinically proven vaccine became available – is significantly higher compared to annual influence vaccination rates (*p* < 0.001). Doctors show a significantly higher willingness to be vaccinated regarding the annual influenza (*p* < 0.001) as well as SARS-CoV2 (*p* < 0.001) when compared to participants from the nursing sector.

We concluded our survey with a section on the personal impact of the COVID-19 pandemic and first evaluated the implication of the pandemic on personal life and mood among medical professionals in Germany. Most participants (44.7%) stated that their private life has “strongly” been restricted by the COVID-19 outbreak (Table 4). Female participants reported a significantly higher level of restriction compared to male participants (*p* < 0.001, Table 4), while this assessment was consistent between professional groups (*p* = 0.142). Interestingly, the impact on private life negatively correlated with participants’ age, meaning that younger participants reported a higher level of restriction (*r*_S_ = −0.132, *p* < 0.001). In terms of personal mood, most participants (48.3%) reported a “negative” influence (Table 4). Again, this negative impression was significantly more prominent among female participants (*p* = 0.002, Figure 3(A), Table 4) and was consistent among professional groups (*p* = 0.524).

**Table 4.** Impact of the COVID-19 pandemic on personal aspects of medical professionals in Germany.

	Not at all (1)	Hardly (2)	Moderately (3)	Strongly (4)	Very strongly (5)	*p* -value
**How much is your private life restricted by the COVID-19 pandemic?**	0.2% (5)	4.2% (113)	20.2% (543)	44.7% (1200)	30.7% (825)	
Female Male	0.1% (2) 0.2% (3)	4.3% (59) 4.1% (53)	18.5% (252) 22.1% (285)	42.2% (575) 47.6% (615)	34.8% (475) 26.0% (336)	<0.001
**How concerned are you about your own health in the context of the COVID-19 pandemic?**	5.8% (155)	31.7% (852)	41.9% (1126)	15.0% (403)	5.6% (150)	
Female Male	5.6% (76) 6.0% (78)	30.8% (420) 32.5% (420)	40.6% (553) 43.3% (559)	16.8% (229) 13.2% (171)	6.2% (85) 5.0% (64)	0.024
Medical doctors Nursing staff Others	6.3% (113) 4.6% (36) 5.0% (6)	34.3% (613) 25.5% (199) 33.3% (40)	43.5% (777) 38.6% (301) 40.0% (48)	11.9% (213) 21.8% (170) 16.7% (20)	3.9% (70) 9.5% (74) 5.0% (6)	<0.001 0.260
**How concerned are you about the health of others in the context of the COVID-19 pandemic?**	0.6% (15)	4.4% (119)	28.9% (777)	46.0% (1236)	20.0% (538)	
Female Male	0.5% (7) 0.6% (8)	3.2% (43) 5.7% (73)	23.8% (325) 34.6% (447)	46.2% (630) 45.9% (592)	26.3% (358) 13.2% (171)	<0.001
Medical doctors Nursing staff Others	0.6% (10) 0.6% (5) 0% (0)	5.5% (99) 2.6% (20) 0% (0)	32.6% (582) 20.0% (156) 32.5% (39)	45.9% (820) 47.2% (368) 40.0% (48)	15.4% (274) 29.6% (231) 27.5% (33)	<0.001 0.004
	Very negative (1)	Negative (2)	Neutral (3)	Positive (4)	Very positive (5)	*p* -value
**How does the COVID-19 pandemic influence your personal mood?**	5.4% (144)	48.3% (1280)	43.7% (1158)	2.5% (67)	0% (1)	
Female Male	6.3% (84) 4.4% (57)	49.8% (665) 46.4% (595)	41.9% (560) 46.1% (591)	2.0% (27) 3.0% (39)	0% (0) 0.1% (1)	0.002

Notes: In case of multiple pairwise comparisons, the level of significance was adjusted by Bonferroni correction. A *p*-value of *p* = 0.017 for three groups and *p* = 0.003 for six groups was considered statistically significant.

Next, we surveyed how concerned medical professionals are about both their own health and the health of others in the context of the COVID-19 pandemic. Most participants described a moderate concern regarding their own health (41.9%) but a strong concern regarding the health of others (46.0%, Table 4). The level of concern regarding their own health and the health of others was significantly higher among female participants (*p* = 0.024 and *p* < 0.001, Table 4) compared to males as well as participants from the nursing sector compared to doctors (both *p* < 0.001, Table 4). Interestingly, the participants’ age correlated positively with the concern about the own health (*r*_S_ = 0.071, *p* < 0.001) but negatively with the concern about the health of others (*r*_S_ = −0.136, *p* < 0.001), meaning that older participants worried more about their own health and younger participants worried more about the health of others.

Once available, a vaccination against SARS-CoV-2 will play a crucial role in achieving herd immunity without tolerating the mortality rate of currently 3.1% (as of 18 April [1]). In terms of the annual influenza, a total of 62.6% stated that they regularly get the annual influenza vaccination. In terms of SARS-CoV-2, 91.1% of participants indicated they would get vaccinated when a clinically proven and safe vaccine became available, which was significantly higher compared to the annual influenza vaccination rate (*p* < 0.001, Figure 3(B)). Importantly, we observed decisive differences in the willingness to be vaccinated among subgroups. As such, doctors showed a significantly higher willingness to be vaccinated regarding influenza (77.2% vs. 42.4%, *p* < 0.001) as well as SARS-CoV2 (94.3% vs. 84.5%, *p* < 0.001) when compared to participants from the nursing sector (Figure 3(B)). The willingness to be vaccinated did not differ between the West and East German federal states (influenza: *p* = 0.397, SARS-CoV-2: *p* = 0.185). Finally, the willingness to get vaccinated for both the annual influenza and SARS-CoV-2 significantly correlated with the participants age (influenza: *r* = 0.069; 95%CI: 0.034–0.103; SARS-CoC2: *r* = 0.084; 95%CI: 0.046–0.119), indicating that older medical professionals are more willing to receive a vaccination.

## Discussion

The ongoing COVID-19 pandemic represents a major global challenge and has pushed healthcare systems around the world to the limit. Although drastic measures in the German healthcare system such as increasing intensive care capacities as well as extensive restrictions of the public life have so far prevented a healthcare collapse, the current situation implicates an enormous burden on medical professionals. Although an emerging number of studies on the impact of the COVID-19 pandemic on healthcare professionals from Asia and other geographical have been published so far (summarized e.g. in [5,6]), the German healthcare system decisively differs from other systems e.g. regarding the high relevance of the outpatient sector provided by general practitioners and specialist [6], which has so far only received limited consideration. Our study is the first to the best of our knowledge to evaluate the perception and impact of the COVID-19 pandemic in terms of private and work-related aspects among medical professionals from both the ambulatory and hospital sector in Germany.

Starting in February 2020, the German healthcare system took significant measures of preparation for the increasing number of COVID-19 patients. While most participants stated that Germany was well prepared for the COVID-19 pandemic, this evaluation was significantly worse among nurses and participants from the ambulatory sector. In line, nurses had significantly more concerns compared to doctors that politically imposed cuts in the health system in recent years might have a negative impact on the COVID-19 pandemic in Germany. These concerns were also more pronounced among medical professionals working on the ICU. With respect to specific COVID-19 training for medical professionals, particularly in the ambulatory sector, participants felt that there were not enough offers in this area. Moreover, based on participants’ statements, COVID-19 training was more often provided for doctors compared to nurses. As early data from Korea have underlined the importance of training for medical professionals in order to protect themselves from SARS-CoV-2 infection [7], it is surprising that only 47.2% of participants reported a specific COVID-19 training and that there were decisive differences with respect to the work site and professional group. Together, these results indicate that, although Germany seems to be well prepared for the pandemic in general, specific attention should be paid to the nursing sector as well as to the ambulatory healthcare sector. In this context it is important to note that on 28 October 2019, the “German regulation for the threshold for nursing staff” came into effect, which e.g. regulates the minimum number of nurses per patient on an intensive care unit (ICU) and aims at assuring high-quality care for ICU patients [8]. However, the majority of hospitals in Germany are currently unable to fill the vacant positions in the nursing sector due to a skills shortage. More than 95% of German hospitals with more than 600 beds were struggling to fill positions for intensive care and standard care nurses in 2019, resulting in a total of 4700 vacant intensive care positions and 12,000 vacant standard care positions [9]. Importantly, these numbers have increased by more than 50% within the last three years [9]. Thus, measures such as expansion of training capacities, financial incentives as well as recruitment from abroad are warranted to improve the situation of the German nursing sector. In terms of financial incentives; however, more than 50% of participants from the nursing sector stated that the COVID-19 pandemic will “hardly” (21.0%) or “not at all” (32.4%) increase the financial income of their professional group.

Shortage of consumable medical equipment such as face masks have been reported in several countries around the globe and endangers health workers worldwide [10,11]. We observed that shortage of consumables was also of relevance in the German healthcare system as over 40% of medical professionals stated that there was a regular (18.1%) or even permanent (16.5%) shortage of consumables at their institution. Importantly, our data suggest that the shortage of medical protective equipment did especially occur in the ambulatory healthcare sector when compared to the hospital sector. Together with our previous data showing that e.g. COVID-19 training is less frequently offered in the ambulatory healthcare sector, these findings argue that the ambulatory healthcare sector should receive more attention during the COVID-19 pandemic in Germany. One could argue that hospitals and especially university and maximum-care hospitals are of higher systemic relevance during the COVID-19 pandemic as they provide highly specialized intensive care medicine including mechanical ventilation or even ECMO therapy. Although these resources are undoubtedly of extreme relevance, they only apply for very small percentage of all COVID-19 patients [2]. As the majority of COVID-19 patients are not hospitalized at all [12], the ambulatory healthcare sector represents an important cornerstone in the treatment landscape of COVID-19 patients and is essential to overcome this pandemic. Thus, supply chains of medical protective equipment in Germany should be amended to ensure sufficient supply of consumable material for the ambulatory sector. In this line of thinking, it is interesting to note that hardly any references regarding the burden on medical professionals in the ambulatory sector in Germany can be found in the international literature, which should trigger further scientific attention.

Herd immunity is essential to eventually contain SARS-CoV-2 dissemination and to prevent future outbreaks. Our study provided information that the willingness of medical professionals to be vaccinated against SARS-CoV-2 (91.1%) is higher compared to the annual influenza (62.6%) and correlated with participants’ age. A potential explanation for a higher willingness to be vaccinated against SARS-CoV-2 compared to influenza is most likely caused by the higher global awareness and mortality rate of SARS-CoV-2, while the positive correlation with age potentially derives from the perceived risk of contracting a serious course of influenza or COVID-19 [13]. In addition, the willingness to be vaccinated was significantly higher among doctors compared to the nursing staff for both viruses (SARS-CoV-2: 94.3% vs. 84.5%, influenza: 77.2% vs. 42.4%), which is in good agreement with data from the RKI showing an influenza vaccination rate in Germany of 76% and 46% among doctors and nurses in the year 2018/2019 [14]. In this context, the RKI previously reported that doctors mainly named “organizational reasons” against the influenza vaccination while nurses reported a general lack of confidence in the vaccine [14].

Besides affecting daily work routine, the ongoing COVID-19 pandemic has a tremendous impact on psychological aspects among healthcare professionals globally. Data from China, where the current pandemic most likely originated, show that 63% of medical professionals experienced a significant level of mental disturbance during the SARS-CoV-2 outbreak with young women being most affected [4,15]. In a different series of health care workers, nurses, women and frontline health care workers reported more severe degrees of mental health symptoms such as depression, anxiety and insomnia than other health care workers [16]. Our data suggest that there is also a significant psychological burden on medical professionals in Germany. Most participants reported that the COVID-19 pandemic negatively influenced their mood (48.3%) and led to a strong (44.7%) or even very strong (33.7%) restriction of private life. Interestingly, both aspects were more prominent among female participants, which is consistent with the data from Asia [15,16]. Interestingly, over 60% of participants had concerns about their own health due to the COVID-19 pandemic and 95% of medical professional stated some concerns regarding the health of others. Again, the level of concern was higher among female participants as well as nurses. The fact that age has been reported as a major risk factor for more severe clinical courses of COVID-19 [17] might be a possible explanation for the positive correlation between participants’ age and the level of concern that we observed in our study.

Our study was limited by some points. First of all, the online survey was conducted within a timeframe of about three weeks. Although this timeframe was specifically chosen as e.g. public restrictions were on the peak during this time, we are unable to provide information about potential longitudinal alterations of perception. Secondly, all results are based on personal statements of medical professionals and thereby do not represent an objective reflection of facts. Importantly, we also did not apply standardized test instruments to evaluate e.g. personal stress or depression and participants were not surveyed about the real frequency of contact with COVID-19 patients as well as existing social support strategies within the work team or at home. Finally, the survey was distributed through various channels and was not actively balanced in relation to the different subgroups, which might implicate an over- or underweighted influence of potential confounders.

Together, this study to best of our knowledge is the first to evaluate the early perception of the SARS-CoV-2 pandemic among medical professionals of both the hospital and ambulatory sector in Germany. Besides establishing an overview on opinion patterns among medical professionals in Germany, we identified decisive differences regarding the status of the German healthcare system e.g. in terms of protective medical equipment and personal attitudes among subgroups. Despite all the caution with which these data are to be interpreted, they may indicate starting points on how medical professionals could be supported in carrying out their important activities and thus mitigate the negative effects of the ongoing and future healthcare challenges.
